# Neurobiological and Behavioral Underpinnings of Perinatal Mood and Anxiety Disorders (PMADs): A Selective Narrative Review

**DOI:** 10.3390/jcm13072088

**Published:** 2024-04-03

**Authors:** Mihaela Oancea, Ștefan Strilciuc, Dan Boitor Borza, Răzvan Ciortea, Doru Diculescu, Dan Mihu

**Affiliations:** 1 Department of Obstetrics and Gynaecology, Iuliu Hatieganu University of Medicine and Pharmacy, 400337 Cluj-Napoca, Romania; 2 Research Center for Functional Genomics, Biomedicine and Translational Medicine, Iuliu Hatieganu University of Medicine and Pharmacy, 400337 Cluj-Napoca, Romania; 3 RoNeuro Institute for Neurological Research and Diagnostic, 400337 Cluj-Napoca, Romania

**Keywords:** perinatal mood and anxiety disorders, postpartum depression, neuroimaging, cognitive function, maternal behavior, brain structure, brain function

## Abstract

Perinatal mood and anxiety disorders (PMADs) profoundly impact maternal and infant health, affecting women worldwide during pregnancy and postpartum. This review synthesizes current research on the neurobiological effects of PMADs, particularly their influence on brain structure, function, and corresponding cognitive, behavioral, and mental health outcomes in mothers. A literature search across PubMed, PsycINFO, and Google Scholar yielded studies utilizing neuroimaging (MRI, fMRI) and cognitive assessments to explore brain changes in PMADs. The key findings indicate significant neurobiological alterations in PMADs, such as glutamatergic dysfunction, neuronal damage, and altered neural connectivity, particularly in postpartum depression (PPD). Functional MRI studies reveal distinct patterns of brain function alteration, including amygdala non-responsivity in PPD, differing from traditional major depressive disorder (MDD). These neurobiological changes are connected with cognitive impairments and behavioral modifications, impacting maternal caregiving. Understanding these alterations is fundamental for developing effective treatments. The findings emphasize the importance of focusing on maternal mental health, advocating for early detection, and personalized treatment strategies to improve maternal and child outcomes.

## 1. Introduction

Perinatal mood and anxiety disorders (PMADs) are prevalent psychological and emotional conditions affecting women during pregnancy and the postpartum period, including depression, anxiety, and other mood disorders [1,2]. A meta-analysis of 291 studies with over 296,000 women from 56 countries reported a global prevalence of postpartum depression (PPD) at 17.7%, revealing notable variation across nations [3]. Anxiety in expectant and new mothers is another dimension of PMADs, with prevalence rates varying depending on the disorders included in the estimates. In some cases, the prevalence of maternal anxiety can be as high as 13% [4]. The ramifications of these disorders extend beyond the immediate mental health of the mother, influencing maternal behavior, brain structure, and function, which, in turn, can have lasting effects on the child’s development and well-being [4,5,6].

Significant neurophysiological alterations occur during pregnancy and postpartum, crucial for maternal and fetal health [7,8,9]. The early postpartum period is accompanied by structural changes in brain regions involved in maternal behavior and motivation, including increases in gray matter volume in the prefrontal cortex, parietal lobes, and midbrain areas such as the hypothalamus, substantia nigra, and amygdala [9]. These modifications are associated with maternal positive perceptions of the baby, suggesting that motherhood is marked by adaptive brain changes that support the development of maternal behaviors and bonding. However, in women with a history of PMADs, these changes may manifest differently, potentially leading to lasting alterations in brain structure and function [10]. Recent advances in neuroimaging and neuroscience have begun to unravel how PMADs affect key brain regions involved in mood regulation and stress response, such as the prefrontal cortex, amygdala, and hippocampus [11,12,13,14,15,16,17]. For instance, Che et al. [15] found increased spontaneous neural activity in the left middle frontal gyrus, left precuneus, left inferior parietal lobule, and left dorsolateral prefrontal cortex (DLPFC) in individuals with peripartum depression, contrasting with decreased activity in the bilateral precentral gyrus and right inferior occipital gyrus compared to healthy controls. Similarly, Wonch et al. [16] observed altered amygdala responsiveness in mothers with depressive symptoms, a region closely interacting with the DLPFC in emotional processing circuits, highlighting how PMADs can distort maternal responses to infant cues and potentially impair mother–infant bonding. Furthermore, Cheng et al. [17] identified distinct patterns of functional connectivity density alterations in women with postpartum depression and anxiety. Understanding these neurobiological changes is essential for recognizing the pathophysiology of PMADs and has significant implications for both cognitive and behavioral outcomes in mothers experiencing them.

Moreover, maternal mental health is intricately linked to maternal–infant bonding, caregiving behaviors, and, ultimately, the child’s psychological and emotional development. Research shows that PMADs can disrupt these critical early interactions, leading to potential long-term developmental challenges for the child [18,19,20,21,22]. There is consistent evidence of small-to-moderate associations between maternal antenatal and postnatal depression and anxiety with poorer offspring social–emotional, cognitive, language, motor, and adaptive behavior development, emphasizing the significance of early detection and intervention [21,23]. These symptoms, if left unaddressed, may persist well into the first year postpartum, significantly impairing the mother’s capacity to form and maintain a nurturing bond with her infant and affecting her relationship with her partner [24].

Given the complexity and intricate nature of PMADs, there is a growing need for a comprehensive synthesis of current research. This narrative review aims to provide a summary of the existing literature on neurobiological and behavioral implications of PMADs during pregnancy and postpartum, integrating results on brain structure, function, and associated cognitive, behavioral, and mental health outcomes in mothers.

## 2. Materials and Methods

### 2.1. Database Search and Strategy

A comprehensive literature search was conducted across PubMed, PsycINFO, and Google Scholar databases. Keywords used in the search included “Perinatal Mood and Anxiety Disorders”, “PMADs”, “postpartum depression”, “postnatal depression”, “perinatal depression”, “perinatal anxiety”, “brain structure”, “brain function”, “neuroimaging”, “cognitive function”, and “behavioral outcomes”. These keywords were combined using Boolean operators to refine the search. No time frame restriction was applied to ensure a comprehensive collection of the relevant literature up to November 2023.

### 2.2. Inclusion and Exclusion Criteria

Inclusion criteria targeted peer-reviewed articles examining brain structural changes in mothers diagnosed with PMADs. The focus was primarily on neuroimaging studies (MRI, fMRI) and those involving cognitive assessments or behavioral measures. A history of PMADs was defined as a diagnosis during pregnancy or the postpartum period.

Exclusion criteria included studies lacking quantitative data on brain structure or function, research involving studies focusing on major depression rather than post-partum depression, or studies that were focused exclusively on the effects of medications on brain structure. In addition, animal studies, systematic reviews, meta-analyses, or research focusing primarily on the impact of PMADs on child development were also excluded from this narrative review.

### 2.3. Study Selection Process

Two independent reviewers screened titles and abstracts for eligibility, followed by a full-text review of selected articles. Disagreements were resolved through discussion or, if necessary, consultation with a third reviewer.

### 2.4. Data Extraction and Synthesis

A total of 101 articles were initially retrieved, and 86 were excluded based on the exclusion criteria during the abstract screening phase. Fifteen articles were then fully reviewed. Further examination of the reference lists within these articles resulted in the identification of five additional relevant studies. Data were extracted systematically, including methodology, sample size, participant characteristics, key findings, and implications from the 19 selected articles. We synthesized the data narratively, focusing on identifying themes and patterns related to the impact of PMADs on brain structure, function, and behavior.

## 3. Results

### 3.1. Brain Spectroscopy and Structural Change

Our review highlights that significant neurobiological changes in brain structure are associated with perinatal mood and anxiety disorders (Table 1). Investigations into brain chemistry reveal glutamatergic dysfunction and neuronal damage in individuals with postpartum depression, as evidenced by significantly lower levels of Glx (Glutamate + Glutamine) and NAA (N-acetylaspartate + N-acetylaspartylglutamate) in the left dorsolateral prefrontal cortex compared to healthy postpartum women [25]. Furthermore, Li et al. [26] identified abnormalities in cortical structures through surface-based morphometry, revealing that patients with PPD exhibit marked alterations in cortical thickness and surface area, particularly in regions implicated in emotional processing and regulation. In a separate study, Li et al. [27] presented a novel perspective on the structural covariance networks of gray matter in individuals with PPD through a graph theoretical analysis. They identified marked alterations in cortical structures and gray matter structural covariance networks, underscoring the profound impact of PPD on the brain’s architecture. These structural changes are supported by findings from Chen et al. [14] and Silver et al. [28] who also observed significant neurobiological changes in individuals with PPD. Silver et al. [28] identified a reduction in fractional anisotropy in the left anterior limb of the internal capsule in patients with PPD, suggesting an alteration in white matter integrity. Chen et al. [14] found increased gray matter volume in the left dorsolateral prefrontal cortex and right precentral gyrus in patients with PPD, correlating with higher Edinburgh Postnatal Depression Scale (EPDS) scores. These structural changes suggest a unique pathological mechanism in PPD, potentially linked to long-term parenting stress [14].

Furthermore, in their investigation into neurotransmitter changes in PPD, McEwen et al. identified elevated Glutamate levels in the medial prefrontal cortex (MPFC) of women with postpartum depression compared to healthy controls. This significant increase in MPFC Glutamate, identified using magnetic resonance spectroscopy, highlights a specific neurochemical alteration associated with PPD, underscoring the impact of extensive hormonal changes during the postpartum period [31]. In addition to the neurochemical and structural changes previously discussed, recent evidence from a resting-state functional magnetic resonance imaging (fMRI) study underscores the alterations in spontaneous neural activity associated with PPD. Che et al. [15] revealed significant differences in the fractional amplitudes of low-frequency fluctuation (fALFF) and regional homogeneity (ReHo) values between patients with PPD and healthy controls, indicating neural dysfunction in PPD. Specifically, their research identified increased spontaneous neural activity in critical areas such as the left middle frontal gyrus, left precuneus, and left dorsolateral prefrontal cortex, alongside decreased activity in the bilateral precentral and right inferior occipital gyrus.

### 3.2. Brain Functional Alterations in PMADs

We identified notable alterations in brain functionality associated with PMADs across the studies reviewed. Silverman et al. observed amygdala non-responsivity in subjects exhibiting greater PPD symptomatology, a finding that contrasts with typical patterns in major depressive disorder (MDD) [29]. Dudin et al. [34] revealed that women with PPD exhibited an enhanced amygdala response to smiling infant pictures compared to non-depressed mothers. This indicates a heightened sensitivity of the amygdala to positive, emotionally salient stimuli in PPD, contrasting with the typical pattern of amygdala responsiveness. Furthermore, Morgan et al. demonstrated that greater depressive severity postpartum correlated with altered neural connectivity, specifically between the right temporoparietal junction (TPJ) and lateral prefrontal cortex. An increase in connectivity was observed between the right TPJ and the anterior medial prefrontal cortex, suggesting difficulties in emotional regulation [38]. Similarly, Bembich et al. [13] identified that mothers observing their newborns in pain displayed significant cortical activation in the left somatosensory and right superior temporal cortex. Importantly, a negative correlation was observed between the activation in the left somatosensory cortex and EPDS scores, indicating reduced cortical responsiveness in mothers with higher depressive symptoms.

Cheng et al. [17] investigated the unique and shared neural disruptions in women experiencing postpartum depression (PPD) and postpartum depression with anxiety (PPD-A) through comprehensive functional connectivity density (FCD) and resting-state functional connectivity (rsFC) analyses. They discovered distinct alterations in long-range FCD, particularly noting weaker connectivity in the right lingual gyrus in PPD patients, contrasted by stronger connectivity in the left ventral striatum for PPD-A patients. Further rsFC analysis revealed common reductions in connectivity between the dorsomedial prefrontal cortex and the ventral striatum across both groups. In the study by Wang et al. [30], significant changes in regional homogeneity (ReHo) were observed in the default mode network of postpartum women with depression. Specifically, there were increases in ReHo in the posterior cingulate and medial frontal gyrus and decreases in the temporal lobes. These findings indicate altered neural activities in areas related to memory and emotional processing in postpartum depression [30]. Interestingly, Schnakenberg et al. [37], in a study involving 157 postpartum women, found no immediate postpartum neuroimaging indicators that could be used as reliable biomarkers for PPD. However, a follow-up at 12 weeks postpartum revealed a significant correlation between EPDS scores and Integrated Local Correlation (LCor) within the left superior medial frontal gyrus, a region implicated in emotional regulation and executive function, suggesting that within this brain region, increased localized connectivity may be linked to the severity of postpartum depressive symptoms. Mao et al. [36] examined the alterations in brain information flow patterns using fMRI and identified significant changes in the preferred direction of information flow among key brain regions implicated in emotion regulation and cognitive processing. There was a disruption in the information flow between the amygdala and regions of the temporal and frontal lobes, critical to emotional processing and mood regulation. Furthermore, they found that these altered information flow patterns strongly correlated with clinical assessments of depression severity, suggesting a potential link between the observed neural changes and the clinical manifestations of PPD. This novel approach highlights the importance of considering not just the activity within specific brain regions but also how information is transmitted between regions in understanding the pathophysiology of PPD.

### 3.3. Behavioral and Cognitive Outcomes Associated PMADs

The postpartum period is marked by complex hormonal changes influencing brain areas involved in social interaction and cognitive processing [40]. Li et al. [39] utilized a Mendelian randomization approach to investigate the potential causal effects of PPD on cognitive impairment. A significant causal relationship was found between PPD and decreased cognitive function, indicating that cognitive impairment is a critical aspect of PPD. Mothers with PPD displayed heightened amygdala responsiveness to positive infant pictures, contrasting with responses in non-postpartum depression. This suggests specific behavioral outcomes in maternal responses to socioemotionally salient stimuli in the postpartum period [34].

Furthermore, Barett et al. [32] highlighted that poorer maternal mood and anxiety were associated with a diminished amygdala response to their own infants’ positive images. This finding implies a direct relationship between maternal emotional state and neural processing of infant cues, which can impact caregiving behavior. In another study, there was an overall increased amygdala response to infant stimuli among mothers with PPD but with reduced amygdala-insular cortex connectivity. This pattern, distinct from mothers without PPD, suggests that PPD may uniquely affect brain responses to infant-related emotional cues, potentially influencing maternal interaction and attachment [16]. The study of Chase et al. identified disrupted connectivity within the default mode network in PPD, particularly between the posterior cingulate cortex (PCC) and the right amygdala [33]. This negative coupling in the PPD group suggests altered processing related to self-relevant thought and social cognition. The role of PCC in self-reflection and empathy implies that these connectivity changes in PPD could affect maternal responsiveness and adaptation to caregiving roles, potentially impacting mother–infant attachment and mentalizing abilities [33].

## 4. Discussion

This narrative review underscores the complexity of neurobiological changes in PMADs, particularly postpartum depression. Rosa et al. identified glutamatergic dysfunction in patients with PPD [25], distinct from the broader spectrum of MDD. The specificity in neurobiological alterations, especially in the amygdala and prefrontal cortex, highlighted by Silverman et al. and Dudin et al. [29,34], underlines the importance of recognizing PPD as a distinct clinical entity. The aberrant amygdala activity in response to emotional stimuli, contrasting with typical patterns in MDD, suggests potential disruptions in maternal responsiveness and emotional processing circuitry [34].

Investigations into postpartum brain function reveal contrasting patterns between typical adaptations and alterations associated with PMADs. Bannbers et al. identified normal neural adaptations in postpartum women without PMADs, such as activation changes in the right inferior frontal gyrus [40]. This contrasts with the findings of Morgan et al. [38], where postpartum depression was associated with altered neural connectivity, particularly decreased connections between the right temporoparietal junction and lateral prefrontal cortex, suggesting difficulties in emotional regulation. Furthermore, one study [41] reported extensive gray matter volume reductions in first-time mothers, specifically in regions linked to the Theory of Mind network. This highlights the significant neuroanatomical changes occurring during the perinatal period and provides a crucial context for understanding the specific alterations seen in PMADs [17]. Understanding these changes is vital for developing tailored interventions to enhance maternal emotional regulation, cognitive processing, and maternal–infant bonding.

The increased GMV in areas like the left dorsolateral prefrontal cortex, associated with the severity of PPD symptoms [14], might reflect cognitive challenges during mother–infant interactions, as discussed by Barrett and Fleming [42], particularly in how mothers process and respond to infant-related stress and demands. This suggests a complex interplay between brain structure and cognitive function in maternal behavior, emphasizing the need for comprehensive approaches to understanding and addressing PMADs, which consider both structural brain changes and cognitive outcomes. The contrasting findings from Bembich et al. [13] and Dudin et al. [34] highlight various ways PPD affects maternal brain function in response to different infant cues. Bembich et al. [13] highlighted that mothers with depressive symptoms showed altered cortical activation when observing their newborns in distress, suggesting an impact of PPD on the neural processing of negative infant-related stimuli. Conversely, Dudin et al. [34] found that women with PPD exhibited an enhanced amygdala response to smiling infant pictures, indicating heightened sensitivity to positive infant cues. These studies suggest that PPD may lead to a complex and nuanced pattern of neural responses to various infant stimuli, both positive and negative. This variability underscores the importance of considering the broad spectrum of emotional and cognitive processing in mothers with PPD, as their reactions to different infant behaviors might be differentially affected by their mood disorder.

Furthermore, the alterations in structural covariance networks identified by Li et al. [27], particularly in regions involved in mood regulation and cognitive processes, parallel the functional connectivity concerns highlighted by Mao et al. [36], reinforcing the fact that the influence of PPD on brain function is deeply intertwined with structural modifications. The novel approach used in these studies underscores the importance of considering not just the activity within specific brain regions but also how information is transmitted between regions in understanding the pathophysiology of PPD.

When discussing the neurobiological and behavioral aspects of PMADs, it is essential to consider the broader implications of these disorders on perinatal outcomes. For instance, Accortt et al. [43] identified a significant association between PMAD diagnoses and adverse perinatal outcomes. Their findings revealed that women with PMADs have a 3.5-fold increase in the likelihood of experiencing complications such as gestational diabetes, preeclampsia, and low birth weight. In addition, Hoffman et al. [44] stressed the importance of the early identification and treatment of PMADs to ensure optimal infant development, citing the far-reaching consequences of these disorders that extend beyond the mother to impact infant neurosynaptic and regulatory development. One study that assessed maternal stress at multiple points during pregnancy identified distinct stress trajectory clusters, with the trajectory of increasing stress in late pregnancy being associated with blunted development of infant negative affect [45]. These trajectory patterns, particularly when involving increased stress in late pregnancy, were also related to stronger neonatal amygdala functional connectivity to regions involved in emotion processing, suggesting that the temporal patterns of maternal stress exposure are key determinants of neurodevelopmental and affective outcomes in infants [45]. Early exposure to maternal stress and mood disorders is associated with altered brain development in infants, as evidenced by changes in amygdala connectivity and responsiveness to emotional stimuli [22,46]. Longitudinal research demonstrates that maternal depression and anxiety significantly affect infants’ social engagement, emotion regulation, and stress reactivity [47]. Studies show that infants of mothers with PMADs have less mature regulatory behaviors, heightened negative emotionality, and increased cortisol reactivity, highlighting altered development trajectories [44,46,48].

### 4.1. Future Research Directions

Findings from structural MRI studies [14,28] open new avenues for research in PMADs. The interplay between structural changes and functional connectivity disturbances [27,36] advances our understanding of the complex pathophysiology of PPD and emphasizes the necessity of addressing structural and functional alterations in devising effective interventions. Future research on PMADs should adopt a multidimensional approach and should perform the following:

- Investigate the longitudinal progression of PMADs from the antenatal period through to the postpartum period and beyond.
- Examine the mechanisms through which hormonal changes impact neurobiological functions and structures, as well as the mother–infant relationship.
- Explore the influence of genetic and epigenetic factors on the development and manifestation of PMADs.
- Assess the efficacy of various treatment modalities, including pharmacological, psychotherapeutic, and digital interventions, in diverse populations to ensure culturally sensitive and accessible care.
- Determine the long-term effects of PMADs on both maternal mental health and child development, with a particular focus on neurodevelopmental and cognitive outcomes.

### 4.2. Summary of Key Findings and Practical Implications

The key findings of our review underscore the significant impact of PMADs on neurobiological changes in the brain. These findings have several practical implications. There is an immediate need for early screening and intervention strategies, as evidenced by the association between PMADs and adverse perinatal outcomes. Enhanced amygdala responsiveness and altered neural connectivity patterns necessitate the development of targeted interventions to improve emotional regulation and cognitive processing in affected mothers. Moreover, understanding the nuanced patterns of neural responses to infant stimuli provides a foundation for interventions aimed at fostering maternal–infant bonding and attachment. Finally, public health initiatives should prioritize increasing awareness of PMADs and the promotion of access to mental health support services for new mothers. Our narrative review emphasizes the necessity of a multidimensional approach to understanding, diagnosing, and treating PMADs. Recognizing the unique neurobiological changes and their impact on maternal behavior and emotional processing is pivotal for maternal and child well-being, guiding future research and clinical practices.

## Figures and Tables

**Table 1:** Summary of key findings (chronological order of publication).

Study	Year	Data Source	Participants	Key Findings	Interpretation
Silverman et al. [29]	2011	fMRI	20 postpartum women	Amygdala non-responsivity to threat-related stimuli in subjects with greater PPD symptomatology.	Indicates distinct emotional processing patterns in PPD compared to MDD.
Wang et al. [30]	2011	fMRI	10 mothers with PPD and 11 healthy mothers	Changes in regional homogeneity in posterior cingulate gyrus, medial frontal gyrus (increased), and temporal lobes (decreased).	Highlights default network (DMN) dysregulation in postpartum depression, affecting memory and emotional processing.
McEwen et al. [31]	2012	Magnetic Resonance Spectroscopy	12 women with PPD, 12 healthy controls	Higher Glutamate levels in the medial prefrontal cortex in women with PPD compared to healthy controls.	Suggests specific neurochemical alterations in PPD, highlighting the impact of hormonal changes during the postpartum period.
Barrett et al. [32]	2012	fMRI	22 postpartum mothers	Reduced amygdala response to positive images of own infants in mothers with poorer maternal experience.	Suggests that maternal mood and anxiety can influence the neural processing of infant cues, impacting caregiving behavior.
Chase et al. [33]	2014	Resting-state fMRI	14 unmedicated postpartum women with major depression and 23 healthy postpartum women	Disrupted connectivity between PCC and right amygdala in PPD, suggesting altered default mode processing.	Implicates impairments in brain networks critical for empathy and social cognition in PPD, influencing mother–infant attachment.
Bembich et al. [13]	2016	Optical topography	30 mothers in early postpartum	Significant cortical activation in the left somatosensory cortex and right superior temporal cortex during infant pain observation. Negative correlation between activation and PPD symptoms.	Highlights impact of maternal emotional states on brain responsiveness to infant cues.
Wonch et al. [16]	2016	fMRI	45 postpartum mothers (28 with PPD, 17 without)	Increased amygdala response in PPD mothers across conditions, with decreased amygdala-insular cortex connectivity when viewing own infants versus other infants.	Indicates altered brain responses to infant-related emotional cues in PPD, potentially affecting maternal interaction and attachment.
Rosa et al. [25]	2017	H-MRS	36 PPD and 25 healthy postpartum women	Lower levels of Glx and NAA in the dorsolateral prefrontal (DLPF) cortex of PPD patients.	Indicates glutamatergic dysfunction and neuronal damage in PPD.
Silver et al. [28]	2018	Structural MRI, DTI	75 pregnant, medication-free women	Lower fractional anisotropy in the left anterior limb of the internal capsule in women with PPD.	Suggests disruption of fronto-subcortical circuits in PPD.
Dudin et al. [34]	2019	fMRI	101 women (mothers/non-mothers with/without depression)	Enhanced amygdala response to smiling infant pictures in women with PPD.	Suggests heightened sensitivity to positive, emotionally salient stimuli in PPD.
Deligiannidis et al. [35]	2019	fMRI, H-MRS	Peripartum and PPD women	Altered DMN connectivity in PPD. Association of cortical GABA concentrations with postpartum resting-state functional connectivity (RSFC).	Peripartum allopregnanolone, through positive allosteric modulatory effects on GABA, might contribute to differences in DMN connectivity in PPD.
Che et al. [15]	2020	fMRI	16 individuals with PPD and 16 healthy controls	Increased spontaneous neural activity in the left middle frontal gyrus, left precuneus, left inferior parietal lobule, and left dorsolateral prefrontal cortex (DLPFC), and decreased activity in the bilateral precentral gyrus and right inferior occipital gyrus in the PPD group. The fractional amplitude of low-frequency fluctuation (fALFF) in the left DLPFC negatively correlated with depression severity.	Suggests that changes in the spontaneous neural activity of these regions are related to emotional responses and severity of depression in PPD.
Mao et al. [36]	2020	Resting-state fMRI	21 patients with PPD and 23 healthy controls	Significant alterations in the preferred information flow direction and index in regions like the amygdala and frontal and temporal lobes. Correlation with depression severity scales.	Suggests disruptions in neural information processing and transmission are central to the pathophysiology of PPD.
Li et al. [26]	2021	Surface-based morphometry	21 drug-naive patients with PPD and 18 healthy postpartum women	Patients with PPD showed thinner cortical thickness in the right inferior parietal lobule, increased surface area in various frontal and temporal regions, and higher mean curvature in the parietal lobules.	These cortical structural alterations, especially in the prefrontal and parietal regions, may serve as markers for assessing PPD severity.
Li. et al. [27]	2021	Graph theoretical analysis of structural MRI	21 drug-naive patients with PPD and 18 healthy postpartum women	Significant alterations in gray matter structural covariance networks in individuals with postpartum depression, highlighting disrupted network connectivity and altered nodal characteristics in specific brain regions.	These alterations point to a complex reconfiguration of brain networks in postpartum depression, which could contribute to the cognitive and emotional symptoms experienced by affected women.
Cheng et al. [17]	2021	fMRI	138 participants (45 unmedicated PPD, 31 PPD-A, 62 healthy postnatal women)	Distinct long-range FCD alterations were found, namely, weaker in the right lingual gyrus for PPD and stronger in the left ventral striatum for PPD-A. Common reductions in connectivity between the dorsomedial prefrontal cortex and the ventral striatum were observed in both PPD and PPD-A.	Disorder-specific and shared alterations in neural connectivity in PPD and PPD-A may inform differential diagnosis and treatment.
Schnakenberg et al. [37]	2021	MRI	157 postpartum women	No significant neuroimaging differences were detected immediately postpartum; correlation between Integrated Local Correlation and postpartum depression severity at 12 weeks.	Brain alterations as biomarkers for PPD are subtle and may be more detectable over time with continuous symptom severity measures.
Morgan et al. [38]	2021	Near-infrared Spectroscopy	23 birth mothers of 12-month-old infants	Altered connectivity between mentalizing regions and affective processing regions in mothers with postpartum depressive symptoms.	Impacts mother–infant interactions and maternal emotional regulation.
Chen et al. [14]	2023	Structural MRI	28 PPD patients and 30 healthy postnatal women (HPW)	Increased GMV in left dorsolateral prefrontal cortex and right precentral gyrus. Correlation with EPDS scores.	Suggests unique structural pathological mechanism in PPD linked to parenting stress.
Li et al. [39]	2023	Genome-wide association study (mendelian randomization)	249.835 women with PPD	Causal relationship between PPD and decreased cognitive function and cognitive performance.	Highlights cognitive impairment as a critical aspect of PPD.

## Data Availability

Data sharing is not applicable to this review as no new data were generated.
